# Macrophages in the inflammatory response to endotoxic shock

**DOI:** 10.1002/iid3.70027

**Published:** 2024-10-10

**Authors:** Xinjie Zhao, Mengjie Wang, Yanru Zhang, Yiyi Zhang, Haojie Tang, Hongyi Yue, Li Zhang, Dan Song

**Affiliations:** ^1^ Key Laboratory for Molecular Genetic Mechanisms and Intervention Research on High Altitude Disease of Tibet Autonomous Region, School of Medicine Xizang Minzu University Xianyang Shaanxi China; ^2^ School of Medicine Xizang Minzu University Xianyang China; ^3^ Affiliated Hospital of Xizang Minzu University Xianyang Shaanxi China

**Keywords:** endotoxic shock, inflammation, lipopolysaccharide, macrophages, NF‐κB/MAPK signaling pathway

## Abstract

**Background:**

Endotoxic shock, particularly prevalent in intensive care units, represents a significant medical challenge. Endotoxin, upon invading the host, triggers intricate interactions with the innate immune system, particularly macrophages. This activation leads to the production of inflammatory mediators such as tumor necrosis factor‐alpha, interleukin‐6, and interleukin‐1‐beta, as well as aberrant activation of the nuclear factor‐kappa‐B and mitogen‐activated protein kinase signaling pathways.

**Objective:**

This review delves into the intricate inflammatory cascades underpinning endotoxic shock, with a particular focus on the pivotal role of macrophages. It aims to elucidate the clinical implications of these processes and offer insights into potential therapeutic strategies.

**Results:**

Macrophages, central to immune regulation, manifest in two distinct subsets: M1 (classically activated subtype) macrophages and M2 (alternatively activated subtype) macrophages. The former exhibit an inflammatory phenotype, while the latter adopt an anti‐inflammatory role. By modulating the inflammatory response in patients with endotoxic shock, these macrophages play a crucial role in restoring immune balance and facilitating recovery.

**Conclusion:**

Macrophages undergo dynamic changes within the immune system, orchestrating essential processes for maintaining tissue homeostasis. A deeper comprehension of the mechanisms governing macrophage‐mediated inflammation lays the groundwork for an anti‐inflammatory, targeted approach to treating endotoxic shock. This understanding can significantly contribute to the development of more effective therapeutic interventions.

## 1 INTRODUCTION

Endotoxic shock is a clinical syndrome. In the present phase, the incidence of endotoxic shock is increasing and is accompanied by a high mortality risk. There are many reasons for inducing endotoxic shock, and involved in the pathogenesis of endotoxic shock in terms of molecular biology are Gram‐negative bacilli that produce lipopolysaccharide (LPS). The primary pathophysiological phenomenon is the malignant compensation of circulatory function accompanied by an irreversible developmental process. The microcirculation is directly involved in the acute inflammatory state of endotoxic shock. Endotoxic shock is characterized by infection, systemic inflammatory response to infection, and the interaction between the host and the infectious agent. It is characterized by the activation of macrophages by LPS from Gram‐negative bacilli and various inflammatory pathways and immune responses. Understanding the pathophysiology of endotoxic shock, especially the molecular mechanisms of macrophages in the inflammatory response to endotoxic shock, can provide a sound basis for the treatment of endotoxic shock.

### 1.1 Introduction to endotoxic shock

#### 1.1.1 Current status of endotoxic shock research

An essential cause of endotoxic shock is infection attacking the host and dysregulating immunity, leading to organ dysfunction and, ultimately, death.^1^ Patients with endotoxic shock are usually treated in the intensive care unit under the current medical infrastructure.^2^ There is substantial morbidity and mortality associated with endotoxic shock. Endotoxic shock has a complex pathophysiology in which LPS binds to complement, antibodies, or other components, resulting in an oxidative burst, endothelial cell dysfunction, a systemic inflammatory response syndrome, and ultimately, coagulation activation, metabolic dysfunction, and multiorgan dysfunction.^3^ Systemic inflammatory response syndrome is characterized by body temperature >38° or <36°, heart rate >90 beats/min or hyperventilation, PaCO_2_ <4.3 kPa, white blood cell count >12 × 10^9^/L or 4 × 10^9^/L, or immature leukocytes >10%.^4^

The intricate etiology of endotoxic shock, stemming from a diverse array of infectious agents, encompasses both Gram‐positive and Gram‐negative bacilli. Notably, extensive research has illuminated a profound link between the pathological evolution of this condition and Gram‐negative bacilli. These bacilli, a prevalent class of pathogenic bacteria, possess a unique cell wall component known as LPS.^5^ As Gram‐negative bacilli proliferate or perish within the body, they liberate significant quantities of LPS, colloquially referred to as endotoxin.^6^ Once it infiltrates the bloodstream, endotoxin prompts a robust immune response, specifically activating macrophages.^7^ This endotoxin serves as a bacterial “messenger,” selectively binding with LPS binding protein (LBP) acute‐phase glycoproteins (LPS‐binding proteins) and subsequently transferring them to cluster of differentiation 14 (CD14) and toll‐like receptor 4 (TLR4) receptors, Toll‐like receptors situated on the macrophage membrane.^8^

CD14 and TLR4 receptors recognize bacterial LPS and induce innate immune responses, autophagy, and cell death, which have important implications for the physiological homeostasis of the organism.^9^ TLR4 activity is closely linked to CD14, and TLR4 proinflammatory receptors that elicit inflammatory responses are well‐known. TLR4 is a member of the toll‐like receptor (TLR) family and is precisely localized on the plasma membrane of immunological and nonimmune cells.^10^

When TLR signaling occurs in macrophages, CD14 on the cell surface regulates its transduction, as the inflammatory response is downregulated in the absence of CD14 and upregulated when CD14 is sufficient, considering the central role of CD14 in TLR4 signaling.^11^ CD14 inhibition reduces the aberrant activity of nuclear factor kappa‐B (NF‐κB) and p38 mitogen‐activated protein kinase (MAPK) inflammatory signaling reversed LPS‐induced autophagic activity and apoptosis.^12^ These results suggest elevated CD14 and TLR4 exacerbate LPS‐induced monocyte/macrophage apoptosis by leading to lysosomal dysfunction and impaired autophagic flux. A close correlation exists between macrophages receiving LPS signaling to induce D14 and TLR4 and activates downstream NF‐κB and MAPK signaling pathways during disease progression in endotoxic shock.

The immune response to endotoxic shock may differ in patients and evolve throughout the disease. Combining biological heterogeneity with biomarkers can help provide individualized treatment.^13^ Early initiation of treatment is generally recommended for patients with endotoxic shock, which continues to show an increasing trend in mortality due to endotoxic shock despite optimal treatment.^14^ Controlling the course of disease involves studying the nature of the disease, such as the course of the physiopathology of endotoxic shock and the molecular mechanisms involved. To study the regulation of NF‐κB and MAPK signaling by CD14 and TLR4 in macrophages involved in the inflammatory response and to provide a theoretical research basis for therapeutic studies of endotoxic shock, provide a forward‐looking perspective.

#### 1.1.2 Functions of macrophages

When cells become necrotic by losing lipid membrane integrity, endogenous damage‐associated molecular patterns (DAMPs) danger signals are released, promoting the response of innate immune cells and mediating the amplification of the inflammatory cascade, ultimately leading to severe organ disease in endotoxin‐shocked patients.^15^ Macrophages are phagocytes with heterogeneity in the innate immune system that play a crucial role in detecting danger signals affecting the functional integrity and pathogenicity of the organism. Macrophages show plasticity, changing their phenotype in response to damage signaled by danger signals and the organism's self‐repair mechanisms.^16^ Macrophages can be present in any tissue, promoting the expression of disease inflammatory signals and regulating reparative functions.

A systemic inflammatory response is elicited during endotoxic shock, which leads to the rapid recruitment of monocytes that evolve into macrophages and the direct production of macrophages at the site of the injured lesion to mediate the course of endotoxic shock disease.^17^ The greater the number of macrophages, the greater the impact on the development of the disease in patients with endotoxic shock.^18^ Thus, macrophages have a significant role in endotoxic shock‐induced tissue damage and increased inflammation expression, downregulating the systemic expression of inflammation in patients with endotoxic shock and recovery of tissue damage.

They play an essential in vivo homeostatic role as giant phagocytes, removing pathogens that cause endotoxic shock and endogenous hazards; this phenomenon maintains the impairment of organ function to endotoxic shock and suppresses the systemic inflammatory response.^19^ The microenvironment is disturbed during endotoxic shock, and macrophages rapidly respond to changes in the organism and intervene in its pathophysiologic functions.^20^ Bone marrow holds immense biological significance, serving as a pivotal source of macrophages.^21^ In the event of infection or inflammation, hematopoietic stem cells residing within the bone marrow exhibit the remarkable capacity to transform into macrophages.^22^ These macrophages then engage actively in the circulation, playing a crucial role in the immune response of tissues. Notably, bone marrow macrophages occupy a central position in the genesis and progression of endotoxic shock.^23^ Upon stimulation by endotoxin, these cells unleash significant quantities of cytokines and inflammatory mediators, a response that has the potential to intensify the inflammatory cascade and ultimately result in tissue injury.^24^ Consequently, therapeutic approaches that specifically target bone marrow macrophages are being viewed as a promising avenue for the development of effective treatments for endotoxin shock.

During this intricate process, they orchestrate a synergistic interplay with a diverse array of factors, encompassing endotoxins, allergenic toxins, cytokines within the autocrine cycle, neuromediators, hemoglobin, and various coagulation factors.^25^ This harmonious interaction ensures the smooth functioning of the system. Stimulation by these factors produces various phenotypes and functional regulation.^26^ Macrophages are found in two distinct subpopulations: First, M1 (classically activated subtype)‐expressing macrophages, also known as the classically activated type, are stimulated by the external danger signal LPS and cause the secretion of inflammatory cytokines interleukin 1‐beta (IL‐1β), interleukin‐6 (IL‐6), and tumor necrosis factor (TNF)‐α. This type amplifies the inflammatory response to endotoxic shock, leading to signs such as organ dysfunction. The other phenotype is the selective M2 (alternatively activated subtype) macrophage, which secretes anti‐inflammatory factors such as interleukin 10 (IL‐10) and transforming growth factor through polarization, reduces the inflammatory response in patients with endotoxic shock, and regulates the immune system to promote patient recovery.^27^

When the macrophage is activated, its phenotypic action eliminates the effects of dangerous cells and phagocytosis of pathogens and intervenes during endotoxic shock disease by polarizing the amount of de‐secreted anti‐inflammatory factors.^28^ Sustained stimulation causes CD14 and TLR activation, leading to the expansion of the proinflammatory cascade. Activation by membrane receptors such as TLRs mediates the increase and maturation of TNF‐α, IL‐6, and IL‐1β with concomitant aberrant activation of NF‐κB or p38 MAPK pathways.^29^

### 1.2 Involvement of macrophages in the inflammatory response to endotoxic shock

#### 1.2.1 Introduction to inflammation

Amplification of inflammatory signals is caused by invading microorganisms and disorders of immune regulation, allergies, physical injuries, and metabolic disorders.^30^ This etiologic view of inflammation provides a classification based on etiologically relevant mechanisms and cause‐specific treatment and prevention.^31^ Macrophages receive exogenous inflammation and elicit a series of genomic responses that continually influence the course of inflammatory diseases.^32^ The genomic era has spawned treatment pathways for treating inflammatory diseases by emphasizing macrophages as the basis of the inflammatory response.

Macrophages produce various cytokines and growth factors for antigenic response, phagocytosis of pathogens, and regulating the immune system in developing inflammatory diseases.^33^ They are stimulated by bacterial LPSs, extracellular promotion of inflammatory protein signaling, and hazardous mediator stimulation. Blocking these danger signals and inflammatory response cells reduces excessive inflammatory expression and allows the host to make some recovery of the injured organism.^34^ Macrophages can be involved in autoregulatory loops during inflammation and play a critical role in initiating, maintaining, and ablating inflammation.^35^

Macrophages have a critical regulatory role in inflammatory mechanisms, macrophages have received increasing attention in a variety of clinical diseases, including endotoxic shock; when endotoxin, microorganisms, and other pathogens invade, the consequent release of many inflammatory factors activates effector cell macrophages, resulting in the release of uncontrolled inflammatory cascade response.^36^ The main stimulus explored here is LPS activation of effector cell macrophages, macrophages generate low‐level proinflammatory factors such as TNF‐α, IL‐6, IL‐1β, and other danger signals, which continually exacerbate the deterioration of the disease process in patients with endotoxic shock.^37^ These biomarkers not only offer the potential for the prompt identification of the pathological phase of endotoxic shock, but they also facilitate aggressive and targeted modulation strategies.^38^ By harnessing the power of these biomarkers, we aim to gain a deeper understanding of the mechanisms underlying endotoxic shock, ultimately paving the way for more effective treatment modalities.

Macrophages undergo various changes in the immune system that can provide behaviors necessary to maintain normal tissue homeostasis during endotoxic shock infections and tissue injury.^39^ By macrophages in the immune system, bidirectional regulation inhibits inflammation; therefore, active and effective intervention in the macrophage changes may point to treatments for inflammatory diseases.^40^ Thus, a better understanding of the mechanisms of macrophage inflammation provides an anti‐inflammatory‐centered foundation for treating viral, bacterial, fungal, and immune system disorders.

#### 1.2.2 Macrophages are involved in inflammatory cytokine regulation

(1) TNF
  In endotoxic shock caused by inflammation, TNF proinflammatory factors are typical in participating in its inflammatory response.^41^ TNF mediates its action through TNF receptor 1 (TNFR1; also known as TNFRI or p55) and TNFR2 (also known as TNFRII or p75).^42^ In macrophages, TNF is expressed as TNFR1 and TNFR2; however, their significance differs, with one having a death structure that leads to the amplification of the inflammatory cascade, and the other does the opposite.^43^
  TNFR receptor expression activates caspase signaling to mediate cellular inflammatory responses, activator protein‐1, and NF‐κB pathways, leading to inflammation and cellular damage.^44^ Due to the differential expression of TNFRs, the TNF signaling pathway is mediated through TNFR1 in most cells, although TNF production occurs in T cells, B cells, and natural killer cells, among others. Macrophages are the primary producers of TNF.^45^
  LPS has long been recognized as a significant stimuli triggering TNF production, for example, in endotoxic shock, where the body is affected by LPS, leading to aberrant activation of CD14 and TLR.^46^ When macrophages receive such signals, they develop two functional phenotypes (M1 or M2), which are present throughout the disease process of toxin shock and continuously stimulate TNFR1 overexpression, promote macrophage polarization toward the MI‐type, increased expression of the inflammatory factor TNF, and activation of the downstream NF‐κB and MAPK signaling pathways, resulting in the deterioration of the condition of patients with endotoxic shock^47^ (Figure 1).
  In an LPS‐induced endotoxic shock experiment in mice, LPS increased the risk of infection and activated the macrophage system MI phenotype, causing the formation of TNF and its massive secretion in the organism. Administration of fructose‐terminal oligosaccharides‐small interfering ribonucleic acids or entacapone, an fructose‐terminated oligosaccharide inhibitor, significantly blocked the polarization response of macrophages toward the MI phenotype and increased macrophages.^48^ The phenotypic function of M2 blocked the expression of inflammatory signals to endotoxic shock and functional impairment of the organism and improved survival in a mouse model of LPS‐induced endotoxic shock.^49^ These findings suggest that inhibition of its MI phenotype and promotion of MII‐type expression could help to control the progression of inflammation, the TNF‐a‐mediated rapid immune response, and the extent and duration of the inhibition, providing a theory for endotoxic shock treatment.
(2) IL‐6
  IL‐6 is a multicellular functional factor regulating the immune response, acute inflammatory phase, and other anti‐infective immunity.^50^ IL‐6 is vital in the cytokine response and is associated with many acute inflammatory diseases, such as classic endotoxic shock.
  IL‐6 participates in the progression of endotoxic shock diseases, promotes inflammatory response, and is secreted and produced in the organism mainly by macrophages.^51^ Rapidly expressed by macrophages upon encountering DAMPs or pathogen‐associated molecular pattern (PAMPs), IL‐6 binds to complexes formed by mlL‐6R and gp130 during classical signaling, IL‐6 binds to slL‐6R contained in serum and tissue fluids during trans transduction signaling, and in the other, IL‐6R directly with and binds to gp130 on nearby localized cells, immediately followed by prompting aberrant activation of NF‐κB and MAPK inflammatory transduction pathways.^52^
  The intricate role of IL‐6 within the innate immune system encompasses a vast array of cellular and molecular interactions. Its effectiveness is vividly showcased by its capacity to stimulate the proliferation and differentiation of immune response‐associated cells, significantly bolstering their functional prowess.^53^ Additionally, IL‐6 synergizes harmoniously with IL‐1 to jointly foster T cell proliferation, a process intricately linked to the upregulation of T cell IL‐2 receptor expression.^54^ This collaborative effort contributes significantly to the augmentation of the body's immunological defenses. Furthermore, IL‐6 plays a pivotal role in the inflammatory response, aiding the body in its effective defense against pathogens.^55^ Elevated levels of IL‐6 are often observed in a broad spectrum of infectious disease scenarios, encompassing viral, bacterial, and fungal infections. This increase is pivotal in activating the immune system and prompting the body to initiate an inflammatory response to combat pathogens.^56^ However, it is crucial to recognize that abnormally elevated levels of IL‐6 may also be intricately linked to the emergence and progression of diverse diseases, such as immune disorders or endotoxic shock.^57^
  Macrophages act as a strategic bridge between innate and adaptive systems. When molecular modules DAMP/PAMPs such as LPS are abnormally expressed during endotoxic shock, macrophages receive these danger signals and induce the abnormal activation of CD14 and TLRs, which prompts macrophages to be activated by the macrophage MI and secrete a large amount of IL‐6 inflammatory factors.^58^ They also activate the downstream NF‐κB and MAPK signaling pathways to the point of deterioration of the condition of endotoxic shock (Figure 1).
  To confirm the mechanism of action of IL‐6 in inflammation‐mediated abnormalities of the innate immune system, experiments using mice with gp130 knock‐in gene mutation found that when IL‐6 was knocked out, IL‐6R transduction was inhibited, and the signaling process of IL‐6 in the organism was blocked; this procedure reduced the abnormal activation of CD14 and TLR, inhibited the macrophage M1 phenotypic response, and regulated the NF‐κB and MAPK signaling pathways on the inflammatory response of the organism, which markedly reduced the inflammatory storm response of LPS on the mouse organism.^59^ Thus, the phenotypic regulation of IL‐6 in macrophages is associated with inflammation and the degree of bacterial infection, significantly elevated more markedly in systemic infections, and its change is a sensitive indicator for diagnosis of endotoxic shock earlier than C‐reactive protein expression.
(3) IL‐1β
  **Figure 1:**
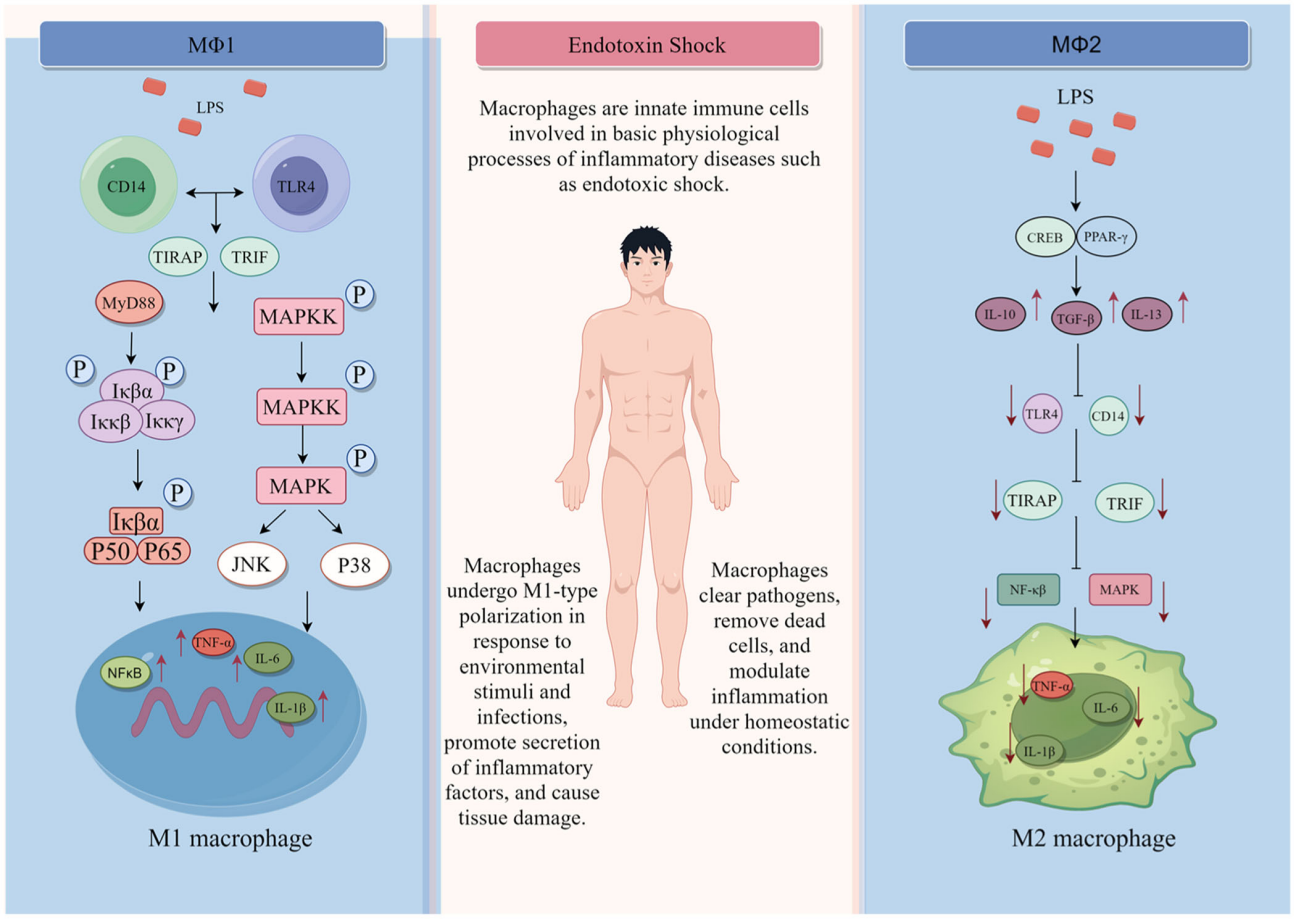
The polarization of macrophages can be divided into classically activated M1 and substitutively activated M2 macrophages. LPS induces CD14 and TLR4 activation to abnormally activate NF‐κB and MAPK signaling pathways and release proinflammatory factors TNF‐α, IL‐6, and IL‐1β, which promote endotoxic shock inflammatory response and lead to disease deterioration. M2 macrophages secrete IL‐10, TGF‐β, and IL‐13 through transformation factors, inhibit the inflammatory response of endotoxic shock, and promote tissue repair. IL‐6, interleukin‐6; IL‐1β, interleukin 1‐beta; LPS, lipopolysaccharide; MAPK, mitogen‐activated protein kinase; NF‐κB, nuclear factor kappa‐B; TNF, and tumor necrosis factor. IL‐10, interleukin 10; IL‐13, interleukin 13; M1, classically activated subtype; M2, alternatively activated subtype; TGF‐β, transforming growth factor.
  LPS stimulates the activation of inflammatory vesicles during cellular infections, and aberrant activation of inflammatory vesicles triggers the maturation of the proinflammatory cytokine IL‐1β, which acts as an important proinflammatory mediator leading to cellular pyroptosis throughout inflammatory diseases such as endotoxic shock.^60^
  First, activation of TLR leads to transcription and translation of pro‐IL‐1β; second, nucleotide‐binding oligomerization domain (NOD)‐like receptor induces IL‐1β processing and release via caspase‐1‐dependent mechanisms while macrophages respond to previous signals exhibiting M1‐type polarization and promoting antigen, followed by aberrant activation of NF‐κB and MAPK signaling pathway transactivation thereby increasing proinflammatory signals and endotoxin disease progression and increased mortality in patients with shock^61^ (Figure 1).
  In a rigorously conducted study seeking to prospectively randomize and stratify mortality risk based on characteristics of macrophage activation syndrome, we delved into the potential significance of interleukin‐1 blockade. The findings of this subgroup analysis revealed a distinct correlation between interleukin‐1 receptor blockade and a substantial enhancement in survival rates among patients suffering from sepsis, coupled with hepatobiliary dysfunction or diffuse intravascular coagulation.^62^ Moreover, in an extensive examination focusing on microglia‐mediated neuroinflammation, we rigorously observed that by delicately balancing the imbalance between TREM2 and TLR4 and equilibrating the downstream NF‐κB activation process, we achieved a marked reduction in the expression level of the M1‐type cell‐specific marker IL‐1β, while significantly augmenting the expression of M2‐type cell markers, including but not limited to interleukin 4 (IL‐4), IL‐10, and CD206.^63^
  This strategic modulation successfully shifted the proinflammatory properties of M1‐type cells to anti‐inflammatory properties of M2‐type cells, thereby enhancing the phosphorylation status of IKKα/β with p65.^64^ Ultimately, this synergistic effect substantially suppressed the inflammatory response triggered by LPS through the mechanism of efficient regulation and control of the inflammatory process by inhibiting the degradation of IκBα and preventing the translocation of p65 into the nucleus.^65^ Despite the challenges of treating sepsis clinically, the rational use of personalized medications with a broad therapeutic spectrum and a high safety profile, effective blockade of the IL‐1 receptor, and improvement of the inflammatory response improve patient survival.^66^

#### 1.2.3 Macrophages regulate inflammatory response signaling pathways

(1) The NF‐κB signaling pathway
  The NF‐κB signaling pathway includes two types. The first is the classical expression pathway through proinflammatory factors, PAMPS, DAMPS, and binding to specific receptors due to ligand activation of the receptor. The activated receptor meets with the TAK1 signaling to make the abnormal activation of the IKK kinase complex, the IKK complex, and the NF‐κB complex to the binding, which leads to the phosphorylation of IκB proteins immediately after the detached IκB inhibitory effect of the NF‐κB transcription complex moves to the nucleus and finally initiates downstream signaling transcription.^67^ When specific receptors such as members of the TNF receptor superfamily, which are NEMO‐independent NF‐κB‐inducible kinase‐mediated, can additionally be mutated to inhibit classical NF‐κB activation and increase the buildup of NF‐κB‐inducible kinases, finally generating aberrant nonclassical NF‐κB signaling.^68^
  LBP interacts with LPS and activates the M1‐type polarization response in macrophages, a potent agonist that promotes the release of inflammatory factors.^69^ When LPS reacts with LBP, it promotes CD14 receptor binding, leading to aberrant activation of the TLR4 receptor, which is recruited in interaction with myeloid differentiation factor 88 family of interface proteins, activating an IKK complex.^70^ The NF‐κB signaling pathway regulates gene expression and can influence various biological processes, regulation of disorders of the innate immune system, downregulation of inflammatory expression, and reduction of response stress [Figure 1].
(2) The MAPK signaling pathway
  The MAPK signaling pathway is a class of evolutionarily highly conserved serine/threonine protein kinases found in most cells in nature, especially in macrophages.^71^ When multiple extracellular stimuli or signaling molecules, physiological stimuli MAPK triggers several pathological responses by transmitting, amplifying, and integrating these signals, such as endotoxic shock, which is the inflammatory response. The MAPK transmission pathway consists of the following: both the MAPK kinase (MAPKK), the MAPKK, and MAPK, with each cascade of responses having specific extracellular lines of initiation and leading to the activation of specific MAPKs upon successive activation of MAPKKKK and MAPKK, signals are transduced through phosphorylation events, and active MAPKs phosphorylate protein targets in the cytoplasm or translocate to the nucleus to phosphorylate many proteins that control gene expression.^72^
  LPS induces the inflammatory response to endotoxic shock, where macrophages undergo an M1‐type polarization response at the onset of endotoxic disease induced by LPS, which stimulates the production of LPS receptor complexes and the formation of a pathway with an inflammatory response cascade.^73^ As TLR4 receptors activate signal transduction within macrophages, leading to MAPKKK, MAPKK, and MAPK, which activate MEK4/7 and c‐Jun N‐terminal kinase (JNK), which can act as a variety of downstream transcription factors and kinases when activated by phosphorylation to increase the inflammatory process of endotoxic shock. They are also activated by phosphorylation of ERK3/6 P38 MAPKK, which, with JNK, induces disease progression in endotoxic shock. Therefore, understanding the polarization response of macrophages can effectively inhibit the inflammatory storm response in endotoxic shock^74^ (Figure 1).

## 2 CONCLUSION

Bacterial infections are a significant threat to human health. Infections by Gram‐negative bacteria elicit an immune response, a significant mediator of which is LPS. LPS‐induced MI‐type activation of macrophages leads to the secretion of many proinflammatory cytokines, such as upregulation of TNF‐a, IL‐6, and IL‐1β expression, and aberrant activation of NF‐κB/MAPK signaling. Excess cytokines may lead to fatal endotoxic shock. Simultaneous effective de‐activation of the M2 type of macrophages can initiate the body's defense program, eliminating the invading bacteria. LPS, a crucial element in the outer membrane of Gram‐negative bacteria, stands as a pivotal factor in triggering endotoxin shock. The heart of its structure, the “virulence center” or lipid‐like A, is integral to its functionality. LPS possesses the unique ability to selectively adhere to LBP, which then facilitates its translocation to the membrane surface of immune cells. Once there, it interfaces intricately with the CD14 protein. This elaborate interaction prompts LPS to activate macrophages, thereby initiating the inflammatory response mechanism. As a result, a diverse network of inflammatory and anti‐inflammatory signaling molecules is triggered, culminating in a complex response system. While effectively mitigating the risks posed by LPS remains a daunting task, we remain steadfast in our pursuit to achieve this goal. We recognize the significant role LPS plays in endotoxic shock and thus its stringent management and control are imperative. By meticulously managing this process, we aim to attenuate the severity of the inflammatory response, ameliorate microcirculatory disorders, and ultimately decrease the likelihood and gravity of shock occurrences.

## AUTHOR CONTRIBUTIONS

**Xinjie Zhao**: Conceptualization; investigation; writing—original draft. **Mengjie Wang**: Formal analysis. **Yanru Zhang**: Conceptualization. **Yiyi Zhang**: Investigation. **Haojie Tang**: Validation. **Hongyi Yue**: Investigation; methodology. **Li Zhang**: Supervision; writing—review and editing. **Dan Song**: Funding acquisition; project administration; supervision; writing—review and editing.

## CONFLICT OF INTEREST STATEMENT

The authors declare no conflict of interest.

## ETHICS STATEMENT

This work was carried out without involving human participants and animals as objects of research.
